# Comparison of Fractional Flow Reserve with Resting Non-Hyperemic Indices in Patients with Coronary Artery Disease

**DOI:** 10.3390/jcdd10020034

**Published:** 2023-01-18

**Authors:** Barbara Zdzierak, Wojciech Zasada, Agata Krawczyk-Ożóg, Tomasz Rakowski, Stanisław Bartuś, Andrzej Surdacki, Artur Dziewierz

**Affiliations:** 1 Clinical Department of Cardiology and Cardiovascular Interventions, University Hospital, 30-688 Krakow, Poland; 2 KCRI, 30-347 Krakow, Poland; 3 Department of Anatomy, HEART-Heart Embryology and Anatomy Research Team, Jagiellonian University Medical College, 33-332 Krakow, Poland; 4 2nd Department of Cardiology, Institute of Cardiology, Jagiellonian University Medical College, 30-688 Krakow, Poland

**Keywords:** intermediate coronary lesions, instantaneous wave-free ratio, fractional flow reserve, resting full-cycle ratio, discordance, physiological assessment

## Abstract

Guidelines recommend using hyperemic (FFR) and non-hyperemic (iFR/RFR) methods of evaluating coronary artery stenoses in patients with coronary artery disease. However, in some cases, achieved results indicating significant ischemia may differ between those methods. Thus, we sought to identify predictors of such a discrepancy. Data were collected on all consecutive patients with chronic coronary syndrome hospitalized between 2020 and 2021. For 279 patients (417 vessels), results for both FFR and iFR/RFR were available. Values of ≤0.80 for FFR and ≤0.89 for iFR/RFR were considered positive for ischemia. Discordant measurements of FFR and iFR/RFR were observed in 80 (19.2%) patients. Atrial fibrillation was the only predictor of the overall FFR and iFR/RFR discordance—OR (95%CI) 1.90 (1.02–3.51); *p* = 0.040. The chance of positive FFR and negative iFR/RFR decreased independently with age—OR (95%CI) 0.96 (0.93–0.99); *p* = 0.024. On the contrary, insulin-treated diabetes mellitus was the predictor of negative FFR and positive iFR/RFR discrepancy—OR (95%CI) 4.61 (1.38–15.40); *p* = 0.013. In everyday clinical practice, iFR/FFR correlates well with FFR. However, discordance between these methods is quite common. Physicians should be aware of the risk of such discordance in patients with atrial fibrillation, advanced age, and insulin-treated diabetes mellitus.

## 1. Introduction

In patients with a high clinical likelihood of coronary artery disease, invasive coronary angiography is reasonable to identify lesions potentially amenable to revascularization [1]. However, the mismatch between coronary stenosis’s angiographic and hemodynamic severity is frequent [2]. Thus, invasive functional assessment should complement coronary angiography, particularly in patients with 50–90% coronary stenosis or multivessel disease [1]. Fractional flow reserve (FFR) is the most commonly used method for this purpose, and measurements are acquired during drug-induced hyperemia [3,4,5,6,7]. Nevertheless, the need to administer hyperemic agents increases procedural costs and may cause discomfort to patients [8,9,10]. Alternative invasive non-hyperemic physiological assessment methods were introduced to address these limitations [3,4]. The first one was the instantaneous wave-free ratio (iFR), a diastolic-only index derived from a standard coronary pressure wire [8,11,12]. Two randomized controlled trials have proved its non-inferiority to FFR in evaluating intermediate lesions [8,12]. In addition, its use in patients with chronic coronary syndromes is advocated by current guidelines [1]. As a result, subsequent non-hyperemic physiological assessment methods were introduced, including resting full-cycle ratio (RFR) [3,4,13]. Despite fundamental differences between iFR and RFR related to the sampling period of the cardiac cycle (wave-free period in diastole for iFR and whole cycle for RFR) used for calculations, RFR was shown to be non-inferior and equivalent to iFR [4,13,14].

There is ample evidence of a good correlation between the hyperemic and non-hyperemic methods of evaluating the significance of coronary artery stenoses [3,4]. Moreover, both methods are recommended to guide coronary revascularization [1]. However, in some cases, achieved results indicating significant ischemia may differ between FFR and iFR/RFR [4,15,16,17]. The knowledge about the factors determining such a discrepancy, especially in all-comers patients, is still limited. Therefore, we sought to identify predictors of the discrepancy between FFR and iFR/RFR in assessing angiographically intermediate coronary artery stenoses.

## 2. Materials and Methods

Data were collected retrospectively on all consecutive patients with chronic coronary syndrome hospitalized at the Clinical Department of Cardiology and Cardiovascular Interventions of the University Hospital in Krakow between 2020 and 2021, in whom invasive physiological assessment of the intermediate coronary lesions was performed. Patients were included regardless of the number of assessed vessels and the method used. Finally, the collected data concerning 381 patients who underwent coronary angiography and in whom the hemodynamic significance of the borderline atherosclerotic stenoses (50–90% diameter stenosis by visual assessment) in the coronary arteries was assessed.

Coronary angiography was performed with the standard radial or femoral approach based on individual operator preferences. All procedures were performed by experienced operators according to a standardized protocol [18]. To assess the hemodynamic significance of the stenosis, FFR, and, during the same procedure, an assessment using another non-hyperemic method was performed. Adenosine was administered as an intracoronary bolus of 100–400 µg for FFR measurement. Depending on the operator’s preferences and device availability, either the iFR or RFR evaluation was performed as part of the non-hyperemic assessment. The measurements were repeated three times, and the mean value was analyzed. As both methods are considered equivalent [13,14], we combined the iFR and RFR results, thus, obtaining information about the result of the non-hyperemic assessment for the entire analyzed group. Then, these results were compared with the FFR results.

Values of ≤0.80 for FFR and ≤0.89 for iFR/RFR were considered positive for ischemia. Depending on the result of the FFR and iFR/RFR assessment, the entire study group was divided into four subgroups, respectively: (1) negative FFR result and negative iFR/RFR result - this group is hereinafter referred to as FFR- | iFR/RFR-; (2) negative FFR result and positive iFR/RFR result - this group is hereinafter referred to as FFR- | iFR/RFR+; (3) positive FFR result and negative iFR/RFR result - this group is hereinafter referred to as FFR+ | iFR/RFR-; (4) positive FFR result and positive iFR/RFR result—this group is hereinafter referred to as FFR+ | iFR/RFR+. Such defined groups were compared considering demographic data, medical data, medical history, and the location of lesions in the coronary circulation. Lesions were identified for which both analyzed methods of the hemodynamic significance gave divergent results, and an attempt was made to identify predictors for such discrepancies.

Categorical variables are presented as numbers and percentages. Continuous variables were expressed as mean, standard deviation (SD), and median and interquartile range (IQR). Differences between groups were compared using analyses of variance (ANOVA) or Pearson’s chi-square test as appropriate. Wilcoxon, each paired test was used for post hoc analysis for multiple comparisons between study groups. The correlation between FFR and iFR/RFR was tested using Pearson’s correlation coefficients. Receiver operating characteristic (ROC) curves were created to assess the optimal cut-off values of FFR to predict iFR/RFR ≤0.89 and iFR/RFR to predict FFR ≤0.80. Factors identified by the stepwise regression model with a *p*-value threshold (0.25 to enter, 0.1 to leave) were included in the multiple regression model. Univariate analyses for factors included in multiple models were presented. Two-sided *p*-values < 0.05 were considered statistically significant. All calculations were performed with JMP^®^, Version 16.1.0 (SAS Institute Inc.).

## 3. Results

Data were collected on 381 patients hospitalized in the Clinical Department of Cardiology and Cardiovascular Interventions of the University Hospital in Krakow, in whom 599 vessels were assessed by FFR and/or iFR/RFR. Of the entire group, patients were selected in whom at least one vessel had been assessed using both the FFR method and the method that did not require hyperemia. This group included 279 patients, in whom 417 vessels were diagnosed. Detailed data on the demographic and clinical characteristics of the included patients and the location of the vessels assessed are presented in Table 1. Most of the evaluated lesions (60%) were located within the left anterior descending artery (LAD). FFR and iFR/RFR varied significantly across the localization of assessed vessels (Table 2).

The study group (vessels) was divided into four prespecified subgroups: FFR- | iFR/RFR- (180 vessels), FFR- | iFR/RFR+ (37 vessels), FFR+ | iFR/RFR- (43 vessels), and FFR+ | iFR/RFR+ (157 vessels). Therefore, discordant measurements were noted in 80 of 417 vessels (19.2%). The analyzed groups differ in sex distribution, arterial hypertension, peripheral vascular disease, and diabetes mellitus treatment. The group in which both FFR and iFR/RFR gave a positive result was older and had a lower estimated glomerular filtration rate (eGFR) compared to the group in which the FFR assessment was positive, and the iFR/RFR assessment was negative. The detailed comparison of the baseline characteristics of the four study subgroups is presented in Table 3.

**Figure 1:**
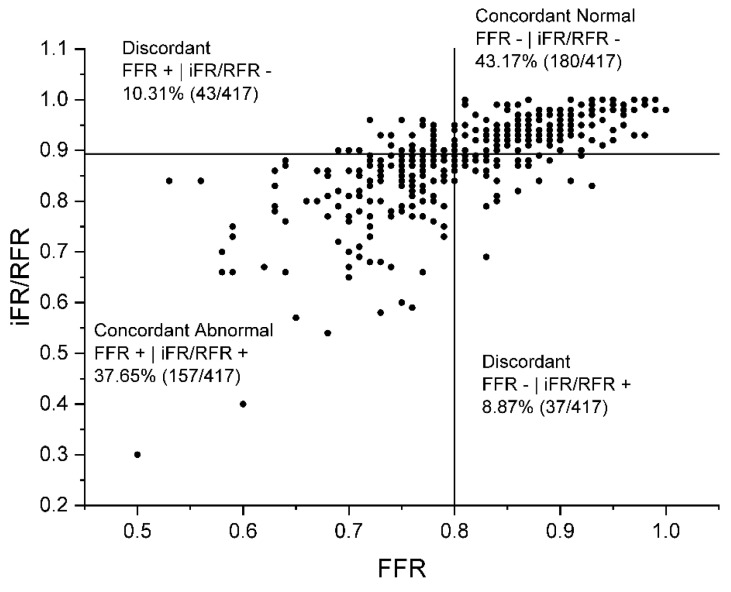
Distribution of lesions according to fractional flow reserve (FFR) and instantaneous wave-free ratio (iFR)/resting full-cycle ratio (RFR) results.

The agreement between the FFR and the iFR/RFR is presented graphically in Figure 1. The diagram shows a high agreement regarding the measurements in which the FFR gave a negative result, while for the lower values of the FFR, the discrepancies between the FFR and the iFR/RFR become slightly clearer. The analysis of the linear correlation of the results obtained using both tested methods shows a similar relationship - the discrepancy between the measurements is slightly smaller for the higher values of FFR and iFR/RFR. In general, the correlation of the results of both tested methods was high (Pearson’s r = 0.71; *p* < 0.0001).

**Figure 2:**
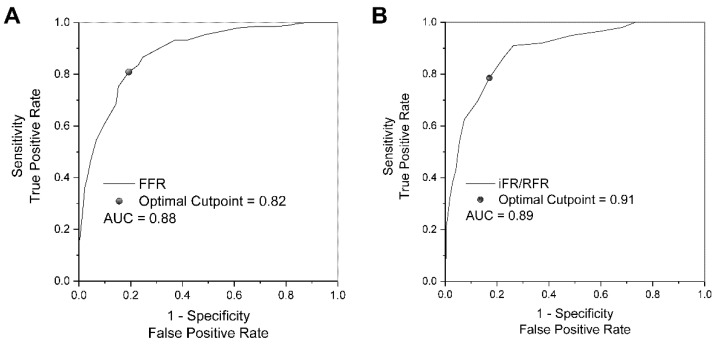
Receiver operating characteristic curves: classification accuracy of FFR (panel **A**) and iFR/RFR (panel **B**). Abbreviations: AUC, the area under the curve; FFR, fractional flow reserve; iFR, instantaneous wave-free ratio; RFR, resting full-cycle ratio.

ROC analysis showed that the optimal cut-off point for FFR to discriminate patients with iFR/RFR ≤0.89 was 0.82. A similar analysis was performed for iFR/RFR, where the optimal cut-off point for distinguishing groups with FFR ≤0.80 was 0.91 (Figure 2).

Univariate analysis of the predictors of FFR+ | iFR/RFR- showed that the chance of being in this group decreased significantly with age. Factors that significantly increased the likelihood of being in the FFR- | iFR/RFR+ group are the treatment of diabetes mellitus with insulin and a low eGFR. However, the impact of eGFR was no longer significant in the multivariate model. The factor that significantly determines the general discrepancy between the measurements was atrial fibrillation, more often seen in discordant groups. Detailed results of the univariate and multivariate analysis are presented in Table 4.

## 4. Discussion

The data presented come from everyday clinical practice and show a high agreement in assessing the significance of angiographically intermediate stenoses in the coronary arteries between the hyperemic and non-hyperemic methods. The discrepancies between those methods were observed in approximately 19.2% of the assessed vessels. A very similar frequency of this phenomenon has previously been reported [4,15,16,19].

The critical limitation of pressure-derived indices is that the pressure gradient is not necessarily a synonym for myocardial ischemia and can be affected by numerous factors [16,17,20,21]. As a result, several clinical and anatomical factors responsible for the discrepancies between the hyperemic and non-hyperemic methods were identified [4]. For instance, a discordance between FFR and iFR was more common in lesions located within the left main coronary artery (LMCA) and the proximal LAD, thus, within vessels supporting large areas of the myocardium [16,19,22]. In our study, only non-LMCA lesions were assessed, and LAD was not identified as an independent predictor of the FFR and iFR/RFR discrepancy. However, LAD was predominant in the group in which both methods confirmed the significance of coronary stenosis. Other factors affecting the agreement between FFR and iFR/RFR measurements include the pattern of coronary disease (focal vs. diffuse), reference vessel diameter, and stenosis severity [4,16,23]. Due to the lack of quantitative coronary angiography analysis data, we could not assess their impact on the occurrence of FFR vs. iFR/RFR discrepancy. In fact, iFR/RFR has been validated in cohorts of patients with intermediate coronary stenoses; thus, the physiological validity of non-hyperemic methods in increasingly stenotic lesions is less established [3,4]. However, severe coronary lesions with >90% stenosis by visual assessment were not scheduled for physiological evaluation in our study.

Insulin-treated diabetes mellitus and eGFR were identified as the risk factors of discordance between FFR- and iFR/RFR+. Similarly, diabetes mellitus was identified as a factor affecting the agreement between FFR and iFR values by several studies [4,24]. This finding might be related to the blunted vasodilation ability due to microvascular dysfunction observed in diabetic patients, which may affect the FFR reliability [17]. Thus, iFR/RFR might be preferred in diabetic patients as its measurement is less prone to vasodilation disturbances [3,17]. In addition, a lower specificity of FFR in predicting ischemia assessed with a single-photon emission computerized tomography in patients with poorly controlled diabetes mellitus was confirmed [25]. On the contrary, in the study of Reith et al., FFR accuracy was not affected by diabetic status and glycemic control [26]. Anatomic optical coherence tomography measurements were better at predicting iFR- than FFR-identified significant lesions in diabetic patients [27]. On the other hand, the equal safety for iFR- and FFR-guided revascularization strategies among patients with diabetes mellitus were confirmed [28]. Decreased eGFR and chronic kidney disease often coexist with diabetes mellitus [29]. Similarly to diabetic patients, FFR can underestimate the true ischemic potential of coronary stenosis in chronic kidney disease patients [17]. For instance, a larger proportion of negative FFR in patients with renal impairment was noted in the FREAK study [30]. In addition, an association between eGFR and the rate of negative FFR was confirmed. Interestingly, lower eGFR values were associated with microvascular impairment and, thus, with the potential risk of suboptimal hyperemia achievement during FFR measurements [30]. In addition to microvascular impairment, the large degree of calcifications might interact with coronary blood flow and result in a blunted hyperemic response [4,17]. These factors may justify the observed relationship between eGFR and FFR vs. iFR/RFR discrepancy.

Age was identified as a predictor of discordance between FFR+ and iFR/RFR-. This discordance was more common in slightly younger patients, and its risk decreased with increasing patient age [15]. Advanced age is frequently associated with comorbidities, including diabetes mellitus, renal impairment, atrial fibrillation, and severe aortic stenosis. These factors may account for the higher risk of FFR and iFR/RFR discrepancies in those patients. In addition, the female gender is more prevalent among patients with advanced age. Notably, the reference vessel diameter is smaller among women than men. Moreover, women more frequently suffer from microvascular disease, which may influence FFR reliability [4,16]. In patients with severe aortic stenosis, FFR and iFR/RFR values might be altered by a falsely low aortic pressure related to the restricted orifice of the aortic valve [17,31]. In addition, a blunted vasodilation ability is suggested in patients with severe aortic stenosis due to myocardial hypertrophy, microvascular dysfunction, and raised left ventricular end-diastolic pressure (LVEDP) [17]. Although increased LVEDP and myocardial hypertrophy might affect the results of both FFR and iFR measurements, iFR seems more reliable in severe aortic stenosis, as its assessment is less likely to be influenced by the blunted vasodilation ability of coronary microcirculation [17,32].

In multivariate analysis, atrial fibrillation was identified as an independent predictor of the overall FFR vs. iFR/RFR discrepancy. This finding might be related to the association of atrial fibrillation with more advanced age. Moreover, atrial fibrillation might be linked with a higher heart rate. Significantly, lower heart rate was associated with a higher risk of a discrepancy between hyperemic and non-hyperemic methods [19]. Interestingly, a recent study indicated an increased beat-to-beat variability of individual iFR measurement in patients with atrial fibrillation compared to patients with sinus rhythm [33]. The reproducibility of iFR was low in patients with atrial fibrillation, leading to the increased reclassification of the lesion. In contrast, FFR variability, reproducibility, and lesion reclassification were comparable between patients with atrial fibrillation and those with sinus rhythm [33]。 Thus, the observed difference in performing FFR vs. iFR/RFR in patients with atrial fibrillation may lead to discordance per se. However, in our study, iFR/RFR measurements were taken three times, and the mean value was used.

The ROC analysis showed that the optimal cut-off value for non-hyperemic methods to identify significant ischemia based on FFR (FFR ≤ 0.80) is 0.91. This value is slightly higher than reported in previous studies [34]. However, other studies reported a higher [15] and a similar optimal cut-off value when comparing FFR with iFR [35]. Similarly, the optimal cut-off value for FFR to detect significant ischemia based on iFR/RFR (iFR/RFR ≤ 0.89) was 0.82. Thus, caution may be required in interpreting borderline FFR values (0.80–0.82), with the possible use of a hybrid approach with iFR/RFR and/or intracoronary imaging. It might be crucial for patients with multiple factors potentially affecting FFR and iFR/RFR discrepancy, i.e., diabetes mellitus, chronic kidney disease, and severe aortic valve stenosis [3,4,17,31]. Further studies are needed to determine how iFR/RFR and FFR cutoff values should be adjusted to account for these individual factors.

The presence of measurement discrepancies may raise concerns about the value of non-hyperemic methods in assessing stenoses located proximally, i.e., stenoses with the most significant impact on the prognosis. However, it was confirmed that, in discordant cases, iFR correlates better with coronary flow reserve than FFR [34]. In addition, an increased risk of adverse outcomes was observed only in patients with concordantly abnormal FFR and iFR and not in the discordant groups [34,36]. However, the prevalence of myocardial perfusion scintigraphy-derived myocardial ischemia in coronary lesions with discordance between FFR and non-hyperemic methods was lower than those with concordantly positive FFR and non-hyperemic methods but higher than those with concordantly negative results [37]. Nevertheless, these findings should be confirmed in further studies. Until their results, closer follow-up and medical management might be justified in discordant patients.

We acknowledge several limitations of this study. The study has a relatively small sample size. No noninvasive assessment of myocardial ischemia was performed; thus, the use of an additional reference method was not possible. Data on the presence of valvular heart disease, central venous pressure, and coronary flow reserve were not collected. The results of quantitative coronary angiography analysis were not available for this study. Long-term follow-up was not conducted to assess clinical endpoints.

## 5. Conclusions

In everyday clinical practice, iFR/FFR correlates well with FFR. However, discordance between these methods of evaluating coronary artery stenoses is quite common. Physicians should be aware of the risk of such discordance in patients with atrial fibrillation, advanced age, and insulin-treated diabetes mellitus.

## Figures and Tables

**Table 1:** Baseline clinical characteristic of the study population. Abbreviations: CABG, coronary artery bypass grafting; COPD, chronic obstructive pulmonary disease; Cx, circumflex artery; Dg, diagonal branch; eGFR, estimated glomerular filtration rate; IQR, interquartile range; LAD, left anterior descending artery; LVEF, left ventricle ejection fraction; Mg, marginal branch; MI, myocardial infarction; PCI, percutaneous coronary intervention; RCA, right coronary artery; SD, standard deviation; TIA, transient ischemic attack.

Value; *n* = 279 Patients		
**Age, years**	**mean (SD)**	67.55 (10.52)
Female	N (%)	70 (25%)
Height, cm	median (IQR)	170.00 (11.00)
Weight, kg	median (IQR)	84.00 (21.88)
Body mass index, kg/m^2^	median (IQR)	28.40 (6.67)
eGFR, mL/min/1.73 m^2^	mean (SD)	76.80 (25.86)
LVEF, %	median (IQR)	52.00 (20.00)
Arterial hypertension	N (%)	242 (87%)
Diabetes mellitus	N (%)	112 (40%)
Diabetes mellitus-treatment	diet, N (%) oral medications, N (%) insulin, N (%)	6 (2%) 63 (23%) 44 (16%)
Atrial fibrillation	N (%)	55 (20%)
Previous MI	N (%)	134 (48%)
Previous PCI	N (%)	141 (51%)
Previous CABG	N (%)	14 (5%)
Peripheral vascular disease	N (%)	42 (15%)
COPD	N (%)	18 (6%)
Stroke/TIA	N (%)	27 (10%)
Current smoker	N (%)	144 (52%)
Dyslipidemia	N (%)	218 (78%)
Number of assessed vessels - per patient	1 (%) 2 (%) 3 (%) 4 (%)	169 (61%) 85 (30%) 22 (8%) 3 (1%)
**value, *n* = 417 vessels**		
Assessed vessels-location	LAD (%) non-LAD (%) Dg (%) Cx (%) Mg (%) RCA (%)	249 (60%) 168 (40%) 17 (4%) 81 (19%) 20 (5%) 50 (12%)

**Table 2:** Distribution of fractional flow reserve (FFR) and instantaneous wave-free ratio (iFR)/resting full-cycle ratio (RFR) across the localization of assessed vessels. * significant differences in post-hoc tests for LAD-RCA; LAD-Cx; LAD-Mg; Dg-RCA; Dg-Cx; Dg-Mg (Wilcoxon Each Pair test or Pearson test with Bonferonni correction), ** significant differences in post-hoc tests for LAD-RCA; LAD-Cx; LAD-Mg; Dg-RCA; Dg-Cx (Pearson test with Bonferonni correction)Abbreviations: Cx, circumflex artery; Dg, diagonal branch; LAD, left anterior descending artery; Mg, marginal branch; RCA, right coronary artery.

	LAD	non-LAD				*p*-Value
		Cx	Mg	Dg	RCA	
FFR, median (Q1–Q3)	0.78 (0.74–0.84)	0.86 (0.79–0.91)				<0.0001
		0.87 (0.8–0.92)	0.88 (0.80–0.91)	0.76 (0.72–0.82)	0.86 (0.82–0.91)	<0.0001 *
FFR ≤ 0.80, N (%)	149 (59.8%)	51 (30.4%)				<0.0001
		21 (25.9%)	5 (25.0%)	13 (76.5%)	12 (24.0%)	<0.0001 *
iFR/RFR, median (Q1–Q3)	0.89 (0.84–0.92)	0.94 (0.88–0.97)				<0.0001
		0.95 (0.89–0.98)	0.93 (0.90–0.98)	0.87 (0.81–0.91)	0.95 (0.91–0.97)	<0.0001 *
iFR/RFR ≤ 0.89, N (%)	145 (58.2%)	49 (29.2%)				<0.0001
		23 (28.4%)	4 (20%)	11 (64.7%)	11 (22%)	<0.0001 **

**Table 3:** Clinical characteristics of the four study groups. * *p* < 0.05 post hoc analysis in relation to the FFR+ | iFR/RFR+ group, ** *p* < 0.05 post hoc analysis in relation to the FFR+ | iFR/RFR- group, *** *p* < 0.05 post hoc analysis in relation to the FFR- | iFR/RFR+ group. Abbreviations: CABG, coronary artery bypass grafting; COPD, chronic obstructive pulmonary disease; Cx, circumflex artery; Dg, diagonal branch; eGFR, estimated glomerular filtration rate; HbA1C, glycated hemoglobin; IQR, interquartile range; LAD, left anterior descending artery; LVEF, left ventricle ejection fraction; Mg, marginal branch; MI, myocardial infarction; PCI, percutaneous coronary intervention; RCA, right coronary artery; SD, standard deviation; TIA, transient ischemic attack.

	FFR- | iFR/RFR- *n* = 180 (43.2%)	FFR- | iFR/RFR + *n* = 37 (8.9%)	FFR+ | iFR/RFR- *n* = 43 (10.3%)	FFR+ | iFR/RFR+ *n* = 157 (37.6%)	*p*-Value
Age, years mean (SD)	67.73 (9.37)	68.68 (12.00)	64.35 (9.96) *	68.13 (10.74)	0.16
Female, N (%)	60 (33.3%) *	10 (27.0%)	6 (14.0%)	30 (19.1%)	0.007
Height, cm median (IQR)	170 (11)	171 (13.25)	172 (11)	170 (11)	0.62
Weight, kg median (IQR)	84 (23)	85 (16.25)	89 (20)	82 (23)	0.75
Body mass index, kg/m^2^ median (IQR)	28.39 (7.56)	28.38 (5.21)	29.04 (5.89)	28.44 (6.68)	0.87
eGFR, mL/min/1.73 m^2^ mean (SD)	77.31 (26.67) **	69.35 (26.84) **	88.74 (26.81) *	77.13 (25.59)	0.010
HbA1c, % median (IQR)	6.5 (2.7)	7.2 (4.4)	8 (4.08)	6.9 (2.3)	0.97
LVEF, % median (IQR)	55 (20)	49.5 (13)	54 (15)	51 (21.5)	0.34
Arterial hypertension, N (%)	159 (88.3%)	33 (89.2%)	33 (76.7%) *	144 (91.7%)	0.042
Diabetes mellitus, N (%)	63 (35.0%)	14 (37.8%)	21 (48.8%)	74 (47.1%)	0.10
Diabetes mellitus treatment - diet, N (%) - oral medications, N (%) - insulin, N (%)	3 (1.7%) 40 (22.2%) 22 (12.2%)	** 1 (2.7%) 3 (8.1%) 10 (27.0%)	0 (0.0%) 18 (41.9%) 3 (7.0%)	5 (3.2%) 38 (24.2%) 31 (19.7%)	0.013
Atrial fibrillation, N (%)	39 (21.7%)	12 (32.4%)	9 (20.9%)	27 (17.2%)	0.23
Previous MI, N (%)	82 (45.6%)	20 (54.1%)	19 (44.2%)	80 (51.0%)	0.62
Previous PCI, N (%)	82 (45.6%)	22 (59.5%)	19 (44.2%)	89 (56.7%)	0.11
Previous CABG, N (%)	12 (6.7%)	1 (2.7%)	1 (2.3%)	5 (3.2%)	0.55
Peripheral vascular disease, N (%)	20 (11.1%) *	5 (13.5%)	4 (9.3%)	37 (23.6%)	0.008
COPD, N (%)	12 (6.7%)	7 (18.9%)	2 (4.7%)	11 (7.0%)	0.16
Stroke/TIA, N (%)	18 (10.0%)	3 (8.1%)	3 (7.0%)	12 (7.6%)	0.88
Current smoker, N (%)	90 (50.0%)	18 (48.6%)	23 (53.5%)	92 (58.6%)	0.41
Dyslipidemia, N (%)	144 (80.0%)	29 (78.4%)	36 (83.7%)	114 (72.6%)	0.50
Assessed vessels - number 1, N (%) 2, N (%) 3, N (%) 4, N (%)	*** 59 (32.8%) 88 (48.9%) 30 (16.7%) 3 (1.7%)	* 14 (37.8%) 13 (35.1%) 4 (10.8%) 6 (16.2%)	21 (48.8%) 13 (30.2%) 9 (20.9%) 0 (0.0%)	75 (47.8%) 56 (35.7%) 23 (14.6%) 3 (1.9%)	<0.0001
Assessed vessels - location LAD, N (%) non-LAD, N (%) - Cx, N (%) - Mg, N (%) - Dg, N (%) - RCA, N (%)	*, ** 77 (42.8%) 103 (57.2%) 54 (30.0%) 14 (7.8%) 2 (1.1%) 33 (18.3%)	23 (62.2%) 14 (37.8%) 6 (16.2%) 1 (2.7%) 2 (5.4%) 5 (13.5%)	27 (62.8%) 16 (37.2%) 4 (9.3%) 2 (4.7%) 4 (9.3%) 6 (14.0%)	122 (77.7%) 35 (22.3%) 17 (10.8%) 3 (1.9%) 9 (5.7%) 6 (3.8%)	<0.0001

**Table 4:** Univariate and multivariate logistic regression analysis for predictors of discordance between FFR and iFR/RFR. Abbreviations: DM, diabetes mellitus; eGFR, estimated glomerular filtration rate; FFR, fractional flow reserve; iFR, instantaneous wave-free ratio; LVEF, left ventricle ejection fraction; RFR, resting full-cycle ratio.

	Univariate Analysis OR (95% Confidence Interval)	*p*-Value	Multivariate Analysis OR (95% Confidence Interval)	*p*-Value
**Predictors of FFR+ | iFR/RFR−**				
Age (per 1 year)	0.96 (0.93–0.99)	0.029	0.96 (0.93–0.99)	0.024
LVEF (per 1%)	1.01 (0.98–1.04)	0.30	1.02 (0.98-1.04)	0.22
**Predictors of FFR− | iFR/RFR+**				
DM treatment (insulin vs. others)	4.64 (1.39–15.48)	0.013	4.61 (1.38–15.40)	0.013
eGFR (per 1 mL/min/1.73 m^2^)	0.98 (0.97–0.99)	0.046	0.99 (0.97–1.02)	0.69
**Predictors of overall FFR and iFR/RFR discordance**				
Age (per 1 year)	0.99 (0.96–1.01)	0.22	0.98 (0.95–1.01)	0.06
LVEF (per 1%)	1.01 (0.99–1.03)	0.28	1.01 (0.99–1.03)	0.15
Atrial fibrillation	1.46 (0.83–2.56)	0.20	1.90 (1.02–3.51)	0.040

## Data Availability

The data presented in this study are available on request from the corresponding author.
